# Cost concentration and resource-use intensity in high-cost HIV/AIDS hospitalizations: evidence from a tertiary infectious disease hospital cohort in China

**DOI:** 10.3389/fpubh.2026.1935687

**Published:** 2026-09-09

**Authors:** Di Shen, Yuwei Wang, Xin Li, Xiduo Yang, Mengfan Yu

**Affiliations:** 1 School of Management, Beijing University of Chinese Medicine, Beijing, China; 2 Medical Insurance Office, Beijing Ditan Hospital, Capital Medical University, Beijing, China

**Keywords:** cost concentration, high-cost hospitalizations, HIV/AIDS, inpatient costs, resource-use intensity

## Abstract

**Background:**

With the expansion of antiretroviral therapy, human immunodeficiency virus/acquired immunodeficiency syndrome (HIV/AIDS) has increasingly become a manageable chronic condition. However, late presentation, opportunistic infections, and critical care can still generate substantial inpatient costs. Mean-based analyses may obscure upper-tail cost concentration. This study quantified HIV/AIDS inpatient cost concentration and resource-use profiles of high-cost hospitalizations.

**Methods:**

We analyzed HIV/AIDS hospitalizations at a tertiary infectious disease hospital between 1 January 2019 and 31 December 2024. High-cost hospitalizations were defined as total inpatient costs at or above the 90th percentile. Cost concentration was assessed using the Lorenz curve, Gini coefficient, and expenditure shares of the top 5 and 10%. Multivariable logistic regression identified factors associated with high-cost hospitalization. Conditional quantile regression examined associations between medical resource-use intensity and costs across quantiles; recentered influence function unconditional 90th-percentile regression was used as a supplementary analysis.

**Results:**

Among 3,240 hospitalizations, the 90th-percentile threshold was RMB 62,683.41. The top 5 and 10% accounted for 23.49 and 35.97% of total inpatient costs; the Gini coefficient was 0.481. Compared with low resource-use intensity, moderate and high resource-use intensity were associated with high-cost hospitalization, with adjusted odds ratios of 18.27 (95% CI, 4.41–75.70) and 90.62 (95% CI, 21.94–374.34), respectively. Prolonged length of stay, a greater number of coded opportunistic or AIDS-defining conditions, and non-local care seeking were also associated with high-cost hospitalization. The estimated cost difference associated with high resource-use intensity was numerically larger at higher cost quantiles, ranging from RMB 18,261.89 at the 25th percentile to RMB 60,178.15 at the 90th percentile.

**Conclusion:**

In this single-center study conducted at a tertiary infectious disease hospital in China, HIV/AIDS inpatient costs were concentrated in a small proportion of high-cost hospitalizations. High resource-use intensity, prolonged stay, and greater coded disease burden characterized high-cost cases, especially in the upper tail. These findings highlight the value of distributional cost analysis for identifying high-resource-use hospitalizations and informing complex case management and hospital resource planning in HIV/AIDS care.

## Introduction

Human immunodeficiency virus/acquired immunodeficiency syndrome (HIV/AIDS) remains a major global public health challenge and continues to threaten improvements in population health and progress toward universal health coverage (1, 2). With the continued expansion of antiretroviral therapy (ART), HIV infection has increasingly shifted from a highly fatal infectious disease to a manageable chronic condition (3). According to WHO estimates, approximately 40.8 million people were living with HIV globally in 2024; 77% were receiving ART, and 73% had achieved viral suppression (4). However, the economic burden of HIV/AIDS remains substantial. In particular, late presentation, severe immune impairment, opportunistic infections, and critical care often require complex inpatient management and intensive medical resource use, which may generate substantial hospitalization costs (5, 6).

In HIV/AIDS hospitalizations, inpatient costs are likely to be unevenly distributed because disease severity, opportunistic infection burden, length of stay, and medical resource use vary widely across cases. A small number of complex hospitalizations may therefore consume a disproportionate share of inpatient resources. Identifying these high-cost cases and their resource-use profiles is important for understanding cost burden beyond average expenditure.

Previous research on HIV/AIDS hospitalization costs can primarily be divided into two categories. The first focuses on the level, composition, and associated factors of inpatient costs, with an emphasis on identifying demographic, clinical, and service-related factors linked to higher hospitalization costs. Existing studies have shown that, even when antiretroviral therapy is provided free of charge, people living with HIV/AIDS may still incur substantial billed total medical charges from diagnostic examinations, laboratory monitoring, treatment of opportunistic infections, complication management, and inpatient services (7–9). Hospital-based studies using medical record or billing data in China further indicate that AIDS stage, prolonged length of stay, impaired immune function, a greater number of coded opportunistic or AIDS-defining conditions or complications, comorbidities, surgical procedures, payment type, and non-local treatment are associated with increased hospitalization costs (7–9). For example, a study of 610 HIV/AIDS inpatients in Nantong found that patients with AIDS had substantially higher mean hospitalization costs and longer hospital stays than those with HIV infection, and that length of stay and number of complications were important factors associated with hospitalization costs (7).

The second category comes from broader health services and health expenditure concentration research, which focuses on whether medical costs are concentrated among a small proportion of patients, commonly described as the high-cost user (HCU) phenomenon (10–12). Previous HCU studies have shown that health care expenditure is rarely evenly distributed across all patients, but often follows a markedly right-skewed and long-tailed distribution, in which a small number of patients consume a substantial share of total medical resources (13–18). Early evidence indicated that high-cost patients have patterns of service use and complications that differ from those of general patients (15). Subsequent studies further showed that HCUs are commonly associated with frequent hospitalizations, longer length of stay, complex disease burden, socioeconomic vulnerability, and intensive use of health services (11, 12, 14, 18). Thus, medical cost burden is not only a question of high average expenditure, but may also reflect the concentration of costs among a small number of complex cases.

However, most existing studies on HIV/AIDS hospitalization costs have relied on mean-based or aggregate approaches and have focused mainly on factors associated with higher costs. Few have examined whether inpatient costs are concentrated among a small number of high-cost hospitalizations or whether the associations between resource use and costs differ across the cost distribution. Although HCU studies have documented cost concentration in general health care settings, evidence remains limited for HIV/AIDS hospitalizations, where disease severity and service use are highly heterogeneous. A distributional perspective is therefore needed to quantify cost concentration and identify the clinical and resource-use profiles of high-cost hospitalizations.

This study used HIV/AIDS hospitalization data from Beijing Ditan Hospital, Capital Medical University, between 2019 and 2024 to address three questions: whether inpatient costs showed marked upper-tail concentration; which patient and service characteristics were associated with high-cost hospitalization; and whether the estimated association between medical resource-use intensity and inpatient costs varied across cost quantiles. To address these questions, we used Lorenz curves and the Gini coefficient to describe cost concentration, multivariable logistic regression to identify factors associated with high-cost hospitalization, and conditional quantile regression together with recentered influence function (RIF) unconditional 90th-percentile regression to examine resource-use patterns in the upper tail of the cost distribution. By applying a distributional perspective, this study aimed to identify high-resource-use HIV/AIDS hospitalizations and provide evidence for characterizing high-resource-use hospitalization profiles and informing complex case management and hospital resource planning. By combining cost-concentration measures with conditional and unconditional distributional regression, this study extends previous mean-based analyses of HIV/AIDS hospitalization costs by identifying factors associated specifically with the upper tail of the cost distribution.

## Materials and methods

### Study design and population

This retrospective observational study used data from the hospital discharge abstract system and hospitalization billing system of Beijing Ditan Hospital, Capital Medical University. Beijing Ditan Hospital is a tertiary infectious disease referral hospital in Beijing and the main hospital supporting the National Center for Infectious Diseases (Beijing). It provides specialized inpatient care for HIV/AIDS and AIDS-related opportunistic infections and receives referrals from Beijing and other regions of China. The source databases covered the institution as a whole, including hospitalization episodes from both the main campus and its branch campus operating during the study period. Because campus identifiers were not retained in the analytic dataset, episodes from the two campuses were pooled and campus-specific analyses were not possible. The cohort therefore included both local and non-local care-seeking episodes treated across the hospital.

We included adult HIV/AIDS hospitalization episodes discharged between 1 January 2019 and 31 December 2024. The unit of analysis was the hospitalization episode rather than the individual patient, and repeat eligible hospitalizations were retained in the primary analysis. A nonmissing hospital information system patient identifier was available for 3,206 episodes, corresponding to 2,059 unique patients; 34 episodes lacked a reliable patient identifier. The first-hospitalization sensitivity analysis was therefore restricted to the 3,206 identifiable episodes and retained the earliest eligible episode for each of the 2,059 patients.

**Figure 1:**
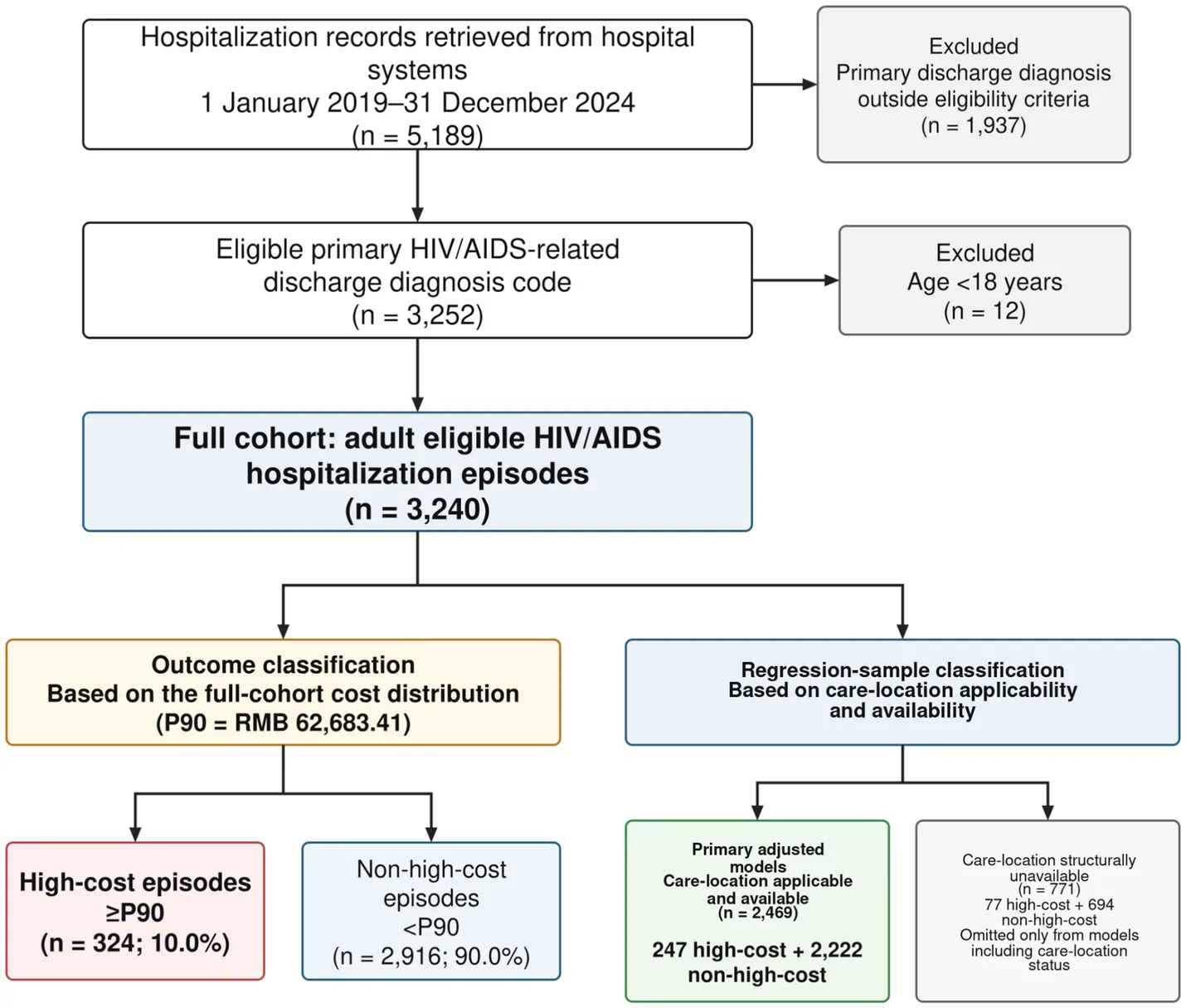
Flow diagram of hospitalization episode selection.

Hospitalization episodes were eligible if they met all of the following criteria: age ≥18 years; admission to the study hospital between January 2019 and December 2024 with completed discharge billing; a primary discharge diagnosis coded using prespecified HIV/AIDS-related ICD-10 or extended ICD-10 codes, including B20.001, B20.101, B20.201, B20.301, B20.401, B20.501, B20.601, B20.701, B20.801, B21.001, B21.101, B21.201, B22.001, B22.003, and B24xx01; and complete information on total inpatient costs, demographic characteristics, and the primary clinical diagnosis. A total of 5,189 hospitalization episodes were initially identified. After excluding 1,937 episodes whose primary discharge diagnoses did not meet the prespecified HIV/AIDS-related ICD-10 criteria and 12 episodes involving patients aged <18 years, 3,240 hospitalization episodes were included in the final analysis (Figure 1). No additional episodes were excluded because of missing information on total inpatient costs, demographic characteristics, or primary clinical diagnosis. Restriction to a primary HIV/AIDS-related discharge diagnosis was applied to improve cohort specificity and identify hospitalization episodes for which HIV/AIDS was the principal reason for admission. Nevertheless, some severe HIV-related hospitalizations may have been primarily coded as tuberculosis, Pneumocystis jirovecii pneumonia, cytomegalovirus disease, sepsis, malignancy, or other complications and therefore may not have been included; this possibility is addressed in the Discussion.

The main outcomes were high-cost hospitalization status and total inpatient costs. Total inpatient costs referred to the billed total medical charges recorded in the hospitalization billing system for a single hospitalization episode, measured in renminbi (RMB). This measure represented the total amount charged for inpatient medical services rather than hospital accounting costs, reimbursement claims, or actual out-of-pocket payments by patients. It included charges for diagnostic examinations, laboratory tests, medications, treatment services, procedures or operations, medical consumables, nursing care, bed charges, and other inpatient medical service items recorded in the billing system. Costs were analyzed in nominal RMB and were not converted to a common price year. Previous studies on high-cost users (HCUs) commonly identify high-cost cases using upper quantiles of the cost distribution, with the top 10% frequently used as a threshold. We therefore used the 90th percentile as the high-cost threshold. This threshold was calculated from the cost distribution of the full cohort of 3,240 hospitalizations. Hospitalizations with total inpatient costs at or above this threshold were defined as high-cost hospitalizations, while the remaining cases were defined as non-high-cost hospitalizations. In this study, the 90th-percentile threshold was RMB 62,683.41. High-cost hospitalization status was used as a binary outcome to describe the characteristics of high-cost cases and to construct the logistic regression model. Continuous total inpatient costs were used to construct the Lorenz curve and calculate the Gini coefficient in the full cohort. Conditional quantile regression and recentered influence function (RIF) unconditional 90th-percentile regression were conducted in the primary analytic sample of 2,469 hospitalization episodes with available care-location information.

The main explanatory variable was medical resource-use intensity, classified as low, moderate, or high according to procedure codes in the discharge abstracts. Low resource-use intensity referred to hospitalizations without invasive procedures. Moderate intensity referred to general invasive procedures, while high intensity referred to intensive care or advanced life-support procedures. This variable was used to characterize the level of medical service and resource use during hospitalization, rather than to infer an independent causal effect.

Covariates included age, sex, payment type, care-location status, prolonged length of stay, and number of coded opportunistic or AIDS-defining conditions. Payment type was categorized as out-of-pocket payment, employee basic medical insurance, resident basic medical insurance, and New Rural Cooperative Medical Scheme (NRCMS)/other payment types for descriptive and full-cohort analyses. Because care-location status was defined from health insurance registration records, the primary regression sample comprised employee- and resident-insured episodes with applicable and available care-location information; employee medical insurance was the reference category. Care-location status was categorized as local or non-local care seeking, with local care seeking as the reference. Prolonged length of stay was defined as a stay exceeding 21 days. The number of coded opportunistic or AIDS-defining conditions was derived from all available discharge diagnosis fields after excluding HIV infection itself. Each prespecified disease category was counted no more than once per hospitalization episode, even if it appeared in multiple diagnosis fields; overlapping terms were consolidated (for example, candidiasis and thrush), non-tuberculous mycobacterial disease was distinguished from tuberculosis, and nonspecific fungal disease was counted only when no specific fungal diagnosis was coded. The resulting continuous, unweighted count was used as an indicator of coded disease burden rather than a severity-weighted clinical measure.

### Statistical analysis

Categorical variables were described as frequencies and percentages. Continuous variables were described as means and standard deviations or medians and interquartile ranges, as appropriate. Between-group comparisons were performed using the *χ*^2^ test, Fisher’s exact test, or the Mann–Whitney *U* test.

Cost concentration was assessed using the Lorenz curve and the Gini coefficient. Multivariable logistic regression was used to examine characteristics associated with high-cost hospitalization. Table 1 reports regression coefficients, standard errors, adjusted odds ratios, 95% confidence intervals, and *p* values. Conditional quantile regression was used to estimate adjusted associations with the conditional 25th, 50th, 75th, and 90th percentiles of total inpatient costs (19). RIF unconditional 90th-percentile regression was used to estimate associations with shifts in the unconditional 90th percentile (20, 21). These approaches address different estimands and their coefficients should not be directly compared. Conditional quantile regression standard errors and confidence intervals were obtained using a heteroskedasticity-robust covariance estimator. For RIF regression, HC1 heteroskedasticity-robust standard errors were obtained from the linear regression of the RIF-transformed outcome. No bootstrap procedure was used.

**Table 1:** Multivariable logistic regression analysis of factors associated with high-cost hospitalization (*N* = 2,469).

Variable	*β*	SE	Adjusted OR	95% CI	*p* value
Age, years	0.011	0.007	1.011	0.997–1.025	0.115
Male sex	−0.521	0.329	0.594	0.311–1.132	0.113
Medical resource-use intensity					
Low resource-use intensity	Reference		1.000		
Moderate resource-use intensity	2.905	0.725	18.269	4.409–75.697	<0.001
High resource-use intensity	4.507	0.724	90.618	21.936–374.342	<0.001
Payment type					
Employee medical insurance	Reference		1.000		
Resident basic medical insurance	−0.273	0.186	0.761	0.529–1.095	0.141
Non-local care seeking	0.477	0.189	1.611	1.112–2.335	0.012
Prolonged length of stay	2.143	0.192	8.529	5.851–12.433	<0.001
Number of coded opportunistic or AIDS-defining conditions	0.312	0.082	1.367	1.164–1.604	<0.001

The analysis included 2,469 hospitalization episodes with available care-location information, including 247 high-cost episodes. The model included age, sex, medical resource-use intensity, payment type, care-location status, prolonged length of stay, and number of coded opportunistic or AIDS-defining conditions. Female sex, low resource-use intensity, employee medical insurance, local care seeking, and no prolonged length of stay were reference groups. OR, odds ratio; CI, confidence interval.

To evaluate the influence of extreme outliers, we conducted a sensitivity analysis after excluding the single hospitalization episode with the highest cost. To assess the influence of repeated hospitalizations from the same patient, we conducted an additional sensitivity analysis restricted to the first eligible hospitalization episode per patient. Because costs were analyzed in nominal RMB and were not converted to a common price year, we further conducted a year-adjusted conditional quantile regression sensitivity analysis by including admission year as a categorical covariate, with 2019 as the reference year.

The primary multivariable analyses were restricted to 2,469 employee- or resident-insured hospitalization episodes for which care-location status was applicable and available; 247 were high-cost episodes. We compared these episodes with the 771 self-payment, NRCMS, or other-payment episodes for which care-location status was structurally unavailable, and fitted a full-cohort sensitivity model that omitted care-location status and retained payment type as four categories. In the full cohort, three high-cost episodes occurred in the low resource-use category; after restriction to the primary analytic sample, two high-cost episodes remained in the low resource-use reference category. Formal linear-programming separation diagnostics using the same analytic sample and covariate specification as the primary logistic model did not identify complete or quasi-complete separation. Nevertheless, because the small number of events in the reference category indicated substantial data sparsity and potential sparse-data bias, we performed Firth penalized logistic regression using the same analytic sample, covariate specification, and reference categories as the primary model. We also performed sensitivity analyses restricted to the first eligible hospitalization per patient and adjusted for calendar year. This study was reported in accordance with the Strengthening the Reporting of Observational Studies in Epidemiology (STROBE) statement and the REporting of studies Conducted using Observational Routinely-collected health Data (RECORD) extension.

All statistical tests were two-sided, and *p* < 0.05 was considered statistically significant. Main analyses were performed using IBM SPSS Statistics version 27.0. Formal complete- and quasi-complete-separation diagnostics were conducted using a linear-programming approach in Python version 3.12.13 with SciPy version 1.17.0. RIF unconditional quantile regression was implemented by generating the recentered influence function of the 90th percentile and fitting a linear regression model using the transformed outcome. Standard errors were obtained from the corresponding regression model.

## Results

### Hospitalization cost distribution and concentration

Of the 5,189 hospitalization episodes initially identified, 3,240 met the eligibility criteria and were included in the final analysis (Figure 1). A nonmissing patient identifier was available for 3,206 episodes, corresponding to 2,059 unique patients; 34 episodes lacked a reliable patient identifier. Care-location information was available for 2,469 hospitalization episodes, of which 247 were high-cost episodes.

**Figure 2:**
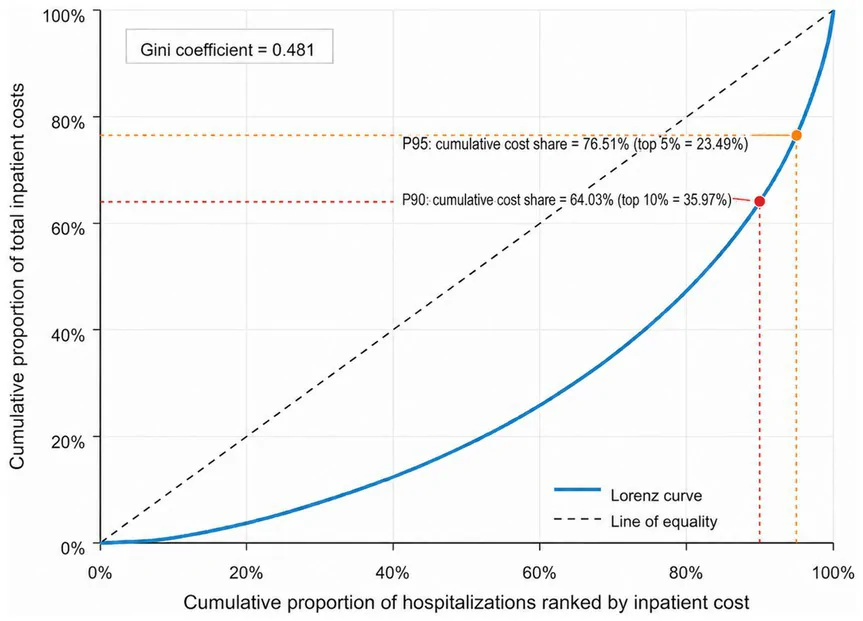
Lorenz curve showing concentration of inpatient costs among HIV/AIDS hospitalizations. P90 and P95 denote the 90th and 95th percentiles of hospitalization episodes ranked by total inpatient costs, corresponding to cumulative cost shares of 64.03 and 76.51%, respectively. Thus, the top 10% and top 5% of hospitalization episodes accounted for 35.97 and 23.49% of total inpatient costs, respectively.

The Lorenz curve showed clear upper-tail concentration of HIV/AIDS inpatient costs. The top 5 and 10% of hospitalizations accounted for 23.49 and 35.97% of total inpatient costs, respectively (Figure 2). The Gini coefficient was 0.481, indicating marked inequality in inpatient costs across hospitalizations.

### Characteristics of high-cost hospitalizations

Compared with the non-high-cost group, the high-cost group showed more intensive resource use and more complex hospitalization profiles. High resource-use intensity was present in 70.7% of high-cost hospitalizations, compared with 13.7% in the non-high-cost group (*p* < 0.001). Prolonged length of stay was also more frequent in the high-cost group (82.1% vs. 17.6%; *p* < 0.001), and the number of coded opportunistic or AIDS-defining conditions was higher (2.00 (2.00, 3.00) vs. 1.00 (1.00, 2.00); *p* < 0.001). The most frequently coded conditions were tuberculosis (25.9%), Pneumocystis jirovecii pneumonia (21.4%), and cytomegalovirus disease (18.7%) (Supplementary Table S5). The proportion of non-local care seeking was higher in the high-cost group than in the non-high-cost group (64.8% vs. 58.1%; *p* = 0.045). Sex and payment type did not differ significantly between groups (Table 2).

**Table 2:** Baseline characteristics and hospitalization cost distribution of HIV/AIDS hospitalizations (*N* = 3,240).

Variable	Overall (*n* = 3,240)	Non-high-cost group (*n* = 2,916)	High-cost group (*n* = 324)	*p*-value
Total inpatient costs, RMB	19,896.88 (11,584.42, 35,748.00)	18,009.61 (10,784.41, 29,954.02)	92,986.20 (72,270.37, 119,919.16)	<0.001
Age, years	38.00 (31.00, 50.00)	39.00 (31.00, 50.00)	36.00 (29.00, 47.00)	0.001
Sex				0.168
Female	298 (9.2)	275 (9.4)	23 (7.1)	
Male	2,942 (90.8)	2,641 (90.6)	301 (92.9)	
Medical resource-use intensity				<0.001
Low	1,298 (40.1)	1,295 (44.4)	3 (0.9)	
Moderate	1,313 (40.5)	1,221 (41.9)	92 (28.4)	
High	629 (19.4)	400 (13.7)	229 (70.7)	
Payment type				0.341
Out-of-pocket payment	484 (14.9)	443 (15.2)	41 (12.7)	
Employee basic medical insurance	1,501 (46.3)	1,352 (46.4)	149 (46.0)	
Resident basic medical insurance	968 (29.9)	870 (29.8)	98 (30.2)	
NRCMS and other payment types	287 (8.9)	251 (8.6)	36 (11.1)	
Care-location status*				0.045
Local care seeking	1,017 (41.2)	930 (41.9)	87 (35.2)	
Non-local care seeking	1,452 (58.8)	1,292 (58.1)	160 (64.8)	
Length of stay, days	13.00 (7.00, 21.00)	11.00 (6.00, 18.00)	34.50 (24.00, 43.00)	<0.001
Prolonged length of stay				<0.001
No	2,462 (76.0)	2,404 (82.4)	58 (17.9)	
Yes	778 (24.0)	512 (17.6)	266 (82.1)	
Number of coded opportunistic or AIDS-defining conditions	1.00 (1.00, 2.00)	1.00 (1.00, 2.00)	2.00 (2.00, 3.00)	<0.001

Values are presented as medians (interquartile ranges) for continuous variables and *n* (%) for categorical variables. Percentages are column percentages. High-cost hospitalizations were defined as total inpatient costs at or above the 90th percentile of the study cohort; the 90th percentile was RMB 62,683.41 in this study. Prolonged length of stay was defined as a length of stay >21 days. *Care-location status was analyzed only among cases with clear health insurance registration location records (*N* = 2,469). *p*-values were derived from the *χ*^2^ test, Fisher’s exact test, or the Mann–Whitney *U* test, as appropriate.

The proportion of high-cost hospitalization increased with resource-use intensity, from 0.2% (3/1,298) in the low-intensity group to 7.0% (92/1,313) in the moderate-intensity group and 36.4% (229/629) in the high-intensity group. High-cost hospitalization occurred in 34.2% (266/778) of hospitalizations with prolonged length of stay, compared with 2.4% (58/2,462) of those without prolonged stay. The proportion was also higher among hospitalizations involving non-local care seeking than among those involving local care seeking [11.0% (160/1,452) vs. 8.6% (87/1,017)].

### Factors associated with high-cost hospitalization

Multivariable logistic regression showed that medical resource-use intensity remained significantly associated with high-cost hospitalization after adjustment for age, sex, payment type, care-location status, prolonged length of stay, and number of coded opportunistic or AIDS-defining conditions. Compared with low resource-use intensity, moderate resource-use intensity was associated with higher odds of high-cost hospitalization (adjusted OR = 18.269, 95% CI, 4.409–75.697; *p* < 0.001), and the corresponding association was stronger for high resource-use intensity (adjusted OR = 90.618, 95% CI, 21.936–374.342; *p* < 0.001) (Table 1).

In addition to resource-use intensity, prolonged length of stay (adjusted OR = 8.529, 95% CI, 5.851–12.433; *p* < 0.001), a greater number of coded opportunistic or AIDS-defining conditions (adjusted OR per additional condition = 1.367, 95% CI, 1.164–1.604; *p* < 0.001), and non-local care seeking (adjusted OR = 1.611, 95% CI, 1.112–2.335; *p* = 0.012) were associated with high-cost hospitalization.

### Associations of resource-use intensity across cost quantiles

Conditional quantile regression showed distributional heterogeneity in the association between medical resource-use intensity and inpatient costs. Compared with low resource-use intensity, moderate resource-use intensity was associated with estimated cost differences of RMB 6,216.59, RMB 7,027.68, RMB 9,506.57, and RMB 13,284.08 at the 25th, 50th, 75th, and 90th conditional percentiles, respectively. The corresponding coefficients for high resource-use intensity were RMB 18,261.89, RMB 23,408.93, RMB 43,998.69, and RMB 60,178.15, respectively (Table 3).

**Table 3:** Conditional quantile regression estimates for factors associated with total inpatient costs across cost quantiles.

Variable	25th percentile (*q* = 0.25)	50th percentile (*q* = 0.50)	75th percentile (*q* = 0.75)	90th percentile (*q* = 0.90)
Age, years	−0.96 (−37.49, 35.56)	33.19 (−11.99, 78.37)	44.00 (−10.55, 98.55)	35.67 (−38.71, 110.06)
Male sex	−3,149.20 (−4,935.69, −1,362.71)	−1,935.08 (−4,044.77, 174.62)	−4,550.37 (−7,000.13, −2,100.60)	−4,107.00 (−7,278.35, −935.64)
Medical resource-use intensity				
Low resource-use intensity	Reference	Reference	Reference	Reference
Moderate resource-use intensity	6,216.59 (5,151.57, 7,281.61)	7,027.68 (5,774.17, 8,281.20)	9,506.57 (8,081.38, 10,931.75)	13,284.08 (11,451.24, 15,116.92)
High resource-use intensity	18,261.89 (16,778.06, 19,745.73)	23,408.93 (21,663.95, 25,153.92)	43,998.69 (42,093.85, 45,903.53)	60,178.15 (57,725.86, 62,630.45)
Payment type				
Employee medical insurance	Reference	Reference	Reference	Reference
Resident basic medical insurance	984.17 (−25.52, 1,993.85)	726.36 (−487.20, 1,939.91)	861.28 (−535.11, 2,257.68)	407.94 (−1,424.95, 2,240.83)
Non-local care seeking	3,976.62 (2,970.86, 4,982.38)	2,199.55 (987.69, 3,411.41)	1,225.96 (−165.76, 2,617.69)	−51.14 (−1,852.33, 1,750.04)
Prolonged length of stay	18,994.49 (17,760.65, 20,228.33)	24,340.72 (22,875.46, 25,805.97)	33,696.89 (32,079.44, 35,314.34)	53,448.48 (51,344.09, 55,552.87)
Number of coded opportunistic or AIDS-defining conditions	1,759.45 (1,296.94, 2,221.96)	2,282.38 (1,724.15, 2,840.62)	3,130.07 (2,482.77, 3,777.37)	3,674.98 (2,864.57, 4,485.39)

Values are *β* coefficients in RMB (95% CI). Conditional quantile regression models were fitted using 2,469 hospitalization episodes with available care-location information. Models adjusted for age, sex, medical resource-use intensity, payment type, care-location status, prolonged length of stay, and number of coded opportunistic or AIDS-defining conditions. Female sex, low resource-use intensity, employee medical insurance, local care seeking, and no prolonged length of stay were reference groups.

The estimated coefficient for high resource-use intensity was numerically larger at higher cost quantiles. Formal statistical comparisons between quantile-specific coefficients were not performed; therefore, these differences should be interpreted as numerical patterns rather than statistically tested differences.

### RIF unconditional 90th-percentile regression

RIF unconditional 90th-percentile regression showed that high resource-use intensity, prolonged length of stay, a greater number of coded opportunistic or AIDS-defining conditions, and non-local care seeking were associated with an upward shift in the unconditional 90th percentile of total inpatient costs (Supplementary Table S2). Compared with low resource-use intensity, the coefficient was RMB 4,553.23 for moderate resource-use intensity and RMB 83,239.88 for high resource-use intensity. The corresponding coefficients were RMB 76,451.29 for prolonged length of stay, RMB 6,753.17 per additional coded condition, and RMB 8,353.23 for non-local care seeking.

### Sensitivity analysis

Cost concentration remained materially unchanged after excluding the single hospitalization episode with the highest cost. Care-location status was applicable and available for the 2,469 employee- or resident-insured episodes and structurally unavailable for the 771 self-payment, NRCMS, or other-payment episodes. The proportion of high-cost hospitalization and the median number of coded opportunistic or AIDS-defining conditions were similar between these groups, whereas age, sex, length of stay, and resource-use intensity differed statistically (Supplementary Table S6). The full-cohort model omitting care-location status and retaining all four payment categories produced materially consistent associations for the principal resource-use variables (Supplementary Table S7). Formal separation diagnostics did not identify complete or quasi-complete separation. However, only two high-cost episodes occurred in the low resource-use reference category in the primary analytic sample, indicating substantial data sparsity. Firth penalized logistic regression produced associations in the same direction as the conventional logistic model, although the estimates for resource-use intensity remained imprecise (Supplementary Table S8).

In the reduced cohort (*N* = 3,239), the top 5 and 10% of hospitalization episodes accounted for 23.19 and 35.70% of total inpatient costs, respectively, close to the full-cohort estimates of 23.49 and 35.97%. This suggests that the observed upper-tail concentration was not driven by a single highest-cost hospitalization episode.

In the sensitivity analysis restricted to the first eligible hospitalization per patient, 2,059 episodes were included and the 90th-percentile threshold was RMB 71,237.79. The directions and statistical conclusions for the principal resource-use variables were materially consistent with the main findings (Supplementary Table S3).

To address potential calendar-year effects, we fitted conditional quantile regression models that included admission year as categorical indicator variables. The principal associations for resource-use intensity, prolonged length of stay, and number of coded opportunistic or AIDS-defining conditions were materially consistent with the main conditional quantile regression results (Supplementary Table S4).

## Discussion

### Principal findings

This study examined the concentration of inpatient costs and the resource-use profiles of high-cost HIV/AIDS hospitalizations from an upper-tail distributional perspective. Three main findings emerged. First, HIV/AIDS inpatient costs showed clear upper-tail concentration: the top 5 and 10% of hospitalizations accounted for 23.49 and 35.97% of total inpatient costs, respectively, and the Gini coefficient was 0.481. This pattern is consistent with previous high-cost user studies showing that medical expenditure is often concentrated among a small proportion of patients (10–14). Our study further shows that such cost concentration also exists among HIV/AIDS hospitalizations, a population with substantial heterogeneity in disease severity, inpatient service use, and resource consumption.

Second, high-cost hospitalizations had identifiable complex inpatient and resource-use profiles. Multivariable analysis showed that high resource-use intensity, prolonged length of stay, and a greater number of coded opportunistic or AIDS-defining conditions were associated with high-cost hospitalization. These findings are broadly consistent with previous HIV/AIDS cost studies linking complex clinical conditions, prolonged hospitalization, opportunistic infection burden, and higher service use to increased hospitalization costs (7–9). They are also consistent with recent systematic reviews showing that AIDS-related illnesses and opportunistic infections remain major contributors to the hospitalization burden among people living with HIV (22, 23). Our study extends this evidence by positioning these factors as characteristics associated with upper-tail costs, rather than only as correlates of higher mean costs.

Third, conditional quantile regression showed that the estimated cost difference associated with high resource-use intensity was numerically larger at higher conditional cost quantiles, ranging from RMB 18,261.89 at the 25th percentile to RMB 60,178.15 at the 90th percentile. Formal comparisons between quantile-specific coefficients were not performed. The supplementary RIF regression further showed an upward shift in the unconditional 90th percentile. These variables were measured contemporaneously with costs and should be interpreted as resource-use profiles rather than causal or admission-time predictors.

### Implications for health economics and policy

From a health economics perspective, the concentration of inpatient costs among a small proportion of HIV/AIDS hospitalizations has implications for health service planning and targeted management. Mean-based cost indicators may be useful for describing the overall economic burden, but they provide limited information for identifying hospitalizations that place disproportionate pressure on inpatient resources. This is particularly important in the context of high-cost users, where a small group of patients may account for a large share of total medical expenditure (13, 16). By quantifying upper-tail cost concentration and linking it to resource-use intensity, prolonged length of stay, and coded disease burden, this study provides empirical evidence for more targeted resource allocation and complex case management.

These findings indicate that average-cost summaries alone may not capture the concentration of inpatient resource use. The observed hospitalization profiles may be useful for retrospective complex-case review, hospital resource planning, and evaluation of care pathways (24, 25). Development of early identification tools would require predictors measured at admission or early in hospitalization and should be assessed in separate prospective studies.

From a health financing and policy perspective, the results identify questions for subsequent evaluation rather than demonstrating effects on efficiency, equity, reimbursement adequacy, or household financial burden. Future studies linking billed charges with reimbursement and out-of-pocket payment data are needed before drawing conclusions about financial protection or affordability. Nevertheless, the concentration of billed charges among hospitalizations involving intensive resource use, prolonged stays, and greater coded disease burden may provide an empirical basis for future evaluation of risk-adjustment and reimbursement arrangements for clinically complex HIV/AIDS cases (26).

For hospital management, discharge abstracts and inpatient records can support retrospective characterization of resource-intensive hospitalization profiles, including intensive procedures, prolonged length of stay, and multiple coded conditions. Because these features were measured during the same episode in which costs accumulated, they should not be interpreted as admission-time predictors. Their practical value lies in complex-case review, resource planning, and generating hypotheses for future admission-based prediction studies.

### Strengths and limitations

This study has several strengths. First, it used discharge abstract and billing data from a tertiary infectious disease hospital over a 6-year period, which allowed us to examine HIV/AIDS inpatient costs in a real-world clinical setting. Second, the study focused explicitly on cost concentration and high-cost hospitalizations, rather than relying only on average costs. This approach is useful for understanding the distributional characteristics of inpatient expenditure. Third, the analysis combined the Lorenz curve, Gini coefficient, multivariable logistic regression, conditional quantile regression, and RIF unconditional 90th-percentile regression. This combination allowed us to examine both the probability of high-cost hospitalization and the heterogeneity of cost associations across the distribution. The sensitivity analysis further showed that the observed upper-tail concentration was not driven by a single highest-cost hospitalization.

These findings should be interpreted within several limitations. First, this was a single-institution, hospitalization-level study at a highly specialized tertiary infectious disease referral hospital. Although the hospital-wide dataset covered both the main and branch campuses, the campuses belonged to the same institution, campus identifiers were unavailable for separate analysis, and the hospital’s referral pathways, broad catchment, and specialized case mix may have enriched the cohort for severe and resource-intensive cases. The results therefore describe this referral institution and should not be treated as nationally representative estimates. Second, repeat admissions were retained because costs were generated at the hospitalization level. A first-hospitalization sensitivity analysis was consistent with the main findings, but the study could not evaluate longitudinal disease progression, outpatient care, adherence, or post-discharge outcomes. Restriction to a primary HIV/AIDS-related discharge diagnosis improved specificity but may also have excluded HIV-related admissions primarily coded as opportunistic infections, malignancies, or other complications. Third, CD4 count, viral load, antiretroviral therapy history, timing of HIV diagnosis, and admission-level severity measures were unavailable, leaving potential residual confounding. Care-location status was structurally unavailable for the 771 self-payment, NRCMS, or other-payment episodes; however, a full-cohort model omitting this variable produced materially consistent principal associations. In addition, only two high-cost episodes occurred in the low resource-use reference category in the primary analytic sample. Although formal diagnostics found no complete or quasi-complete separation and Firth analysis was consistent with the conventional model, the resource-use estimates remained imprecise and potentially affected by sparse-data bias. Fourth, costs were analyzed in nominal RMB, although adjustment for admission year did not materially alter the findings. Finally, resource-use intensity, length of stay, procedures, coded conditions, and costs were measured within the same hospitalization. The coded-condition count was unweighted, and diagnosis timing could not distinguish conditions present at admission from those identified during the stay. These variables therefore characterize contemporaneous high-cost hospitalization profiles rather than causal or admission-time predictors. The study also lacked reimbursement, out-of-pocket expenditure, socioeconomic, cost-effectiveness, and appropriateness-of-care data and cannot support conclusions about financial protection, affordability, efficiency, or equity.

## Conclusion

HIV/AIDS inpatient costs showed clear upper-tail concentration in this tertiary infectious disease hospital cohort. High-cost hospitalizations were characterized by high medical resource-use intensity, prolonged length of stay, and greater coded disease burden, with larger numerical cost differences observed at higher quantiles of the cost distribution. These findings suggest that distributional cost analysis can support the characterization of high-resource-use hospitalizations and inform complex case management and hospital resource planning in HIV/AIDS care.

## Data Availability

The data are not publicly available because they were derived from the hospital discharge abstract and hospitalization billing systems and are subject to patient privacy protections and institutional data-governance requirements. De-identified data may be available from the corresponding authors upon reasonable request, subject to approval by the participating institution and the relevant ethics committee and compliance with applicable data-security requirements.
