# Supplementary material for: Cost concentration and resource-use intensity in high-cost HIV/AIDS hospitalizations: evidence from a tertiary infectious disease hospital cohort in China
Source: Front Public Health. 2026 Sep 9;14:1935687. doi: 10.3389/fpubh.2026.1935687 (PMC13597741; doi:10.3389/fpubh.2026.1935687)
Supplement: Supplementary file 1 [file Data_sheet_1.pdf]

Supplementary Table S1. Definitions and model coding of study variables

Variable	Definition or classification criterion	Model specification
Total inpatient costs	Billed total medical charges recorded for one hospitalization episode, measured in nominal RMB	Continuous outcome for distributional analyses
High-cost hospitalization	Total inpatient costs at or above the full-cohort 90th percentile (RMB 62,683.41)	Binary outcome; 0 = non-high-cost, 1 = high-cost
Age	Age at admission, years	Continuous variable
Sex	Sex recorded in the discharge abstract	Female reference
Medical resource-use intensity	Classified from procedure codes and level of inpatient resource use	Categorical; low reference
Low resource-use intensity	No invasive diagnostic or therapeutic procedures; routine treatment, basic nursing, and general examinations	Reference
Moderate resource-use intensity	General invasive diagnostic or therapeutic procedures, such as thoracentesis, abdominal paracentesis, endoscopy, and central venous catheterization	Indicator variable
High resource-use intensity	Intensive care or advanced life-support procedures, including invasive mechanical ventilation, continuous renal replacement therapy, extracorporeal membrane oxygenation, intensive care treatment, or massive transfusion support	Indicator variable
Payment type	Billing category: self-payment, employee basic medical insurance, resident basic medical insurance, or NRCMS/other payment types	Primary N=2,469 model: employee reference and resident comparison; full-cohort models: four categories, self-payment reference
Care-location status	Whether hospitalization occurred within or outside the health insurance registration location	Binary; local reference; used only when registration-location information was available
Prolonged length of stay	Length of stay exceeding the full-cohort 75th percentile (>21 days)	Binary; no reference
Number of coded opportunistic or AIDS-defining conditions	Unweighted count of prespecified coded disease categories from all available discharge diagnosis fields after excluding HIV infection itself; each category counted at most once per episode and overlapping terms consolidated	Continuous count; not a severity-weighted score
HIV/AIDS diagnosis codes	Primary discharge diagnosis included prespecified ICD-10 B20–B24 codes and extensions	Eligibility criterion; not a regression covariate

Note: Medical resource-use intensity was used to characterize the level of inpatient medical service use and resource utilization. It should not be interpreted as a randomly assigned treatment exposure or used

to infer an independent causal effect. Categorical variables were entered into regression models as dummy variables.

Supplementary Table S2. RIF unconditional quantile regression estimates for the 90th percentile of total inpatient costs

Variable	β coefficient (RMB)	Robust SE	95% CI	P value
Age, years	236.71	155.00	-67.09–540.51	0.127
Male sex	-10,825.85	6,756.82	-24,069.22–2,417.51	0.109
Medical resource-use intensity				
Low resource-use intensity	Reference			
Moderate resource-use intensity	4,553.23	2,256.76	129.98–8,976.48	0.044
High resource-use intensity	83,239.88	7,889.74	67,775.99–98,703.77	<0.001
Payment type				
Employee medical insurance	Reference			
Resident basic medical insurance	-4,714.35	3,980.20	-12,515.53–3,086.84	0.236
Non-local care seeking	8,353.23	3,954.45	602.50–16,103.95	0.035
Prolonged length of stay	76,451.29	7,001.94	62,727.48–90,175.10	<0.001
Number of coded opportunistic or AIDS-defining conditions	6,753.17	1,905.51	3,018.36–10,487.97	<0.001

Note: N=2,469 hospitalization episodes. The dependent variable was the RIF-transformed value corresponding to the unconditional 90th percentile of total inpatient costs within the primary analytic sample. HC1 heteroskedasticity-robust standard errors are reported. Female sex, low resource-use intensity, employee medical insurance, local care seeking, and no prolonged length of stay were reference groups.

Supplementary Table S3. Sensitivity analysis restricted to the first eligible hospitalization per patient

Variable	β	SE	Adjusted OR	95% CI	P value
Age, years	0.006	0.007	1.006	0.992–1.021	0.376
Male sex	-0.218	0.333	0.804	0.419–1.544	0.513
Medical resource-use intensity					
Low resource-use intensity	Reference		1.000		
Moderate resource-use intensity	2.876	1.018	17.741	2.412–130.472	0.005
High resource-use intensity	4.517	1.014	91.571	12.558–667.700	<0.001
Payment type					
Self-payment	Reference		1.000		
Employee medical insurance	0.538	0.268	1.713	1.013–2.896	0.045
Resident basic medical insurance	0.638	0.280	1.894	1.093–3.280	0.023
NRCMS and other payment types	0.573	0.356	1.774	0.884–3.563	0.107
Prolonged length of stay	1.759	0.200	5.804	3.925–8.584	<0.001
Number of coded opportunistic or AIDS-defining conditions	0.178	0.080	1.194	1.021–1.398	0.027

Note: This sensitivity analysis included the first eligible hospitalization for each of 2,059 patients with a nonmissing patient identifier. High-cost hospitalization was defined using the restricted-cohort 90th-percentile threshold (RMB 71,237.79). Payment type was entered as four categories with self-payment as the reference; care-location status was omitted.

Supplementary Table S4. Calendar-year-adjusted conditional quantile regression sensitivity analysis

Variable	25th percentile	50th percentile	75th percentile	90th percentile
Moderate resource-use intensity	6,046.67 (4,985.43, 7,107.91)	7,065.28 (5,834.04, 8,296.51)	8,834.23 (7,391.70, 10,276.76)	12,138.45 (10,279.18, 13,997.72)
High resource-use intensity	17,882.93 (16,397.52, 19,368.33)	23,286.86 (21,576.68, 24,997.05)	44,211.19 (42,283.35, 46,139.04)	60,783.88 (58,273.72, 63,294.03)
Prolonged length of stay	18,234.33 (16,991.65, 19,477.01)	24,395.42 (22,953.27, 25,837.58)	33,898.79 (32,248.44, 35,549.14)	51,758.68 (49,617.61, 53,899.74)
Number of coded opportunistic or AIDS-defining conditions	1,701.90 (1,239.06, 2,164.75)	2,229.27 (1,680.76, 2,777.77)	2,778.45 (2,118.84, 3,438.05)	3,663.43 (2,810.98, 4,515.88)

Note: N=2,469 hospitalization episodes. Models included all primary-model covariates and categorical indicator variables for admission year (2019 reference). Values are β coefficients in RMB (95% CI); the table displays the principal resource-use variables.

Supplementary Table S5. Frequencies of major coded opportunistic or AIDS-defining conditions among HIV/AIDS hospitalization episodes

Coded opportunistic or AIDS-defining condition	Hospitalization episodes, n (%)
Tuberculosis	838 (25.9)
Pneumocystis jirovecii pneumonia	692 (21.4)
Cytomegalovirus disease	606 (18.7)
Candidiasis	332 (10.2)
Cryptococcosis	247 (7.6)
Kaposi sarcoma	144 (4.4)
Lymphoma	105 (3.2)
Talaromyces (formerly Penicillium) marneffei infection	53 (1.6)
Non-tuberculous mycobacterial infection	30 (0.9)
Aspergillosis	27 (0.8)

Note: Data are presented as number of hospitalization episodes and percentage. Each prespecified coded opportunistic or AIDS-defining disease category was counted no more than once per hospitalization episode, even if it appeared in multiple diagnosis fields. Overlapping terms were consolidated; non-tuberculous mycobacterial disease was distinguished from tuberculosis, and nonspecific fungal disease was counted only when no specific fungal diagnosis was coded. Because individual conditions differ substantially in clinical severity and treatment complexity, the resulting count was unweighted. The timing of diagnosis could not be determined from the discharge abstract data; therefore, it was not possible to distinguish conditions present at admission from those identified during hospitalization.

Supplementary Table S6. Comparison of hospitalization characteristics according to care-location information availability

Variable	Applicable and available care-location information (N=2469)	Structurally unavailable care-location information (N=771)	P value
Age, years, median (IQR)	39 (32–50)	37 (30–48)	<0.001
Male sex, n (%)	2281 (92.4)	661 (85.7)	<0.001
Length of stay, days, median (IQR)	12 (6–21)	13 (7–21)	0.039
Number of coded opportunistic or AIDS-defining conditions, median (IQR)	1 (1–2)	1 (1–2)	0.097
High-cost hospitalization, n (%)	247 (10.0)	77 (10.0)	1.000
Payment type, n (%)			—
Employee medical insurance	1501 (60.8)	0 (0.0)	
Resident basic medical insurance	968 (39.2)	0 (0.0)	
Self-payment	0 (0.0)	484 (62.8)	
NRCMS and other payment types	0 (0.0)	287 (37.2)	
Resource-use intensity, n (%)			0.037
Low	1015 (41.1)	283 (36.7)	
Moderate	995 (40.3)	318 (41.2)	
High	459 (18.6)	170 (22.0)	

Note: Data are presented as median (interquartile range) or number (percentage). Continuous variables were compared using the Mann–Whitney U test, and categorical variables were compared using the χ^2 test. Care-location status was derived from health insurance registration records and was applicable and available for employee- and resident-insured episodes; it was structurally unavailable for self-payment and other payment types. Because payment type determined whether care-location status was applicable, no inferential P value was calculated for payment type.

Supplementary Table S7. Full-cohort logistic regression sensitivity analysis without care-location status

Variable	β	SE	Adjusted OR	95% CI	P value
Age, years	0.002	0.006	1.002	0.990–1.014	0.779
Male sex	-0.328	0.289	0.720	0.408–1.270	0.257
Medical resource-use intensity					
Low resource-use intensity	Reference		1.000		
Moderate resource-use intensity	2.736	0.594	15.426	4.817–49.398	<0.001
High resource-use intensity	4.273	0.593	71.766	22.455–229.359	<0.001
Payment type					
Self-payment	Reference		1.000		
Employee medical insurance	0.361	0.221	1.434	0.931–2.210	0.102
Resident basic medical insurance	0.308	0.235	1.361	0.859–2.155	0.189
NRCMS and other payment types	0.450	0.292	1.569	0.885–2.781	0.123
Prolonged length of stay	2.094	0.165	8.121	5.882–11.213	<0.001
Number of coded opportunistic or AIDS-defining conditions	0.204	0.068	1.226	1.073–1.400	0.003

Note: This sensitivity analysis included all 3,240 hospitalization episodes. Care-location status was omitted. Payment type was entered as four categories with self-payment as the reference. Female sex, low resource-use intensity, and no prolonged length of stay were the other reference groups.

Supplementary Table S8. Firth penalized logistic regression sensitivity analysis for high-cost hospitalization

Variable	β	SE	Adjusted OR	95% CI	P value
Age, years	0.011	0.007	1.011	0.997–1.025	0.115
Male sex	-0.528	0.326	0.590	0.312–1.117	0.105
Medical resource-use intensity					
Low resource-use intensity	Reference		1.000		
Moderate resource-use intensity	2.688	0.646	14.706	4.144–52.180	<0.001
High resource-use intensity	4.274	0.645	71.809	20.298–254.043	<0.001
Payment type					
Employee medical insurance	Reference		1.000		
Resident basic medical insurance	-0.270	0.185	0.763	0.532–1.096	0.144
Non-local care seeking	0.471	0.188	1.601	1.108–2.315	0.012
Prolonged length of stay	2.121	0.190	8.338	5.743–12.104	<0.001
Number of coded opportunistic or AIDS-defining conditions	0.309	0.081	1.362	1.162–1.597	<0.001

Note: N=2,469 hospitalization episodes. Within this primary analytic sample, only two high-cost episodes occurred in the low resource-use reference category; three such events occurred in the full cohort. Formal linear-programming separation diagnostics did not identify complete or quasi-complete separation. Firth penalized logistic regression was performed to address potential sparse-data bias arising from the small number of events in the reference category. The covariate specification and reference groups matched those of the primary model.

Supplementary Table S9. Complete results of conditional quantile regression models for total inpatient costs

Variable	Quantile	β coefficient (RMB)	SE	95% CI	P value
Age, years	Q25	-0.96	18.64	-37.49–35.56	0.959
	Q50	33.19	23.05	-11.99–78.37	0.150
	Q75	44.00	27.83	-10.55–98.55	0.114
	Q90	35.67	37.95	-38.71–110.06	0.347
Male sex	Q25	-3,149.20	911.47	-4,935.69–-1,362.71	<0.001
	Q50	-1,935.08	1,076.37	-4,044.77–174.62	0.072
	Q75	-4,550.37	1,249.88	-7,000.13–-2,100.60	<0.001
	Q90	-4,107.00	1,618.04	-7,278.35–-935.64	0.011
Moderate resource-use intensity	Q25	6,216.59	543.38	5,151.57–7,281.61	<0.001
	Q50	7,027.68	639.55	5,774.17–8,281.20	<0.001
	Q75	9,506.57	727.14	8,081.38–10,931.75	<0.001
	Q90	13,284.08	935.12	11,451.24–15,116.92	<0.001
High resource-use intensity	Q25	18,261.89	757.06	16,778.06–19,745.73	<0.001
	Q50	23,408.93	890.30	21,663.95–25,153.92	<0.001
	Q75	43,998.69	971.86	42,093.85–45,903.53	<0.001
	Q90	60,178.15	1,251.17	57,725.86–62,630.45	<0.001
Resident basic medical insurance	Q25	984.17	515.15	-25.52–1,993.85	0.056
	Q50	726.36	619.16	-487.20–1,939.91	0.241
	Q75	861.28	712.45	-535.11–2,257.68	0.227
	Q90	407.94	935.15	-1,424.95–2,240.83	0.663
Non-local care seeking	Q25	3,976.62	513.14	2,970.86–4,982.38	<0.001
	Q50	2,199.55	618.30	987.69–3,411.41	<0.001
	Q75	1,225.96	710.06	-165.76–2,617.69	0.084
	Q90	-51.14	918.97	-1,852.33–1,750.04	0.956
Prolonged length of stay	Q25	18,994.49	629.51	17,760.65–20,228.33	<0.001
	Q50	24,340.72	747.58	22,875.46–25,805.97	<0.001
	Q75	33,696.89	825.23	32,079.44–35,314.34	<0.001
	Q90	53,448.48	1,073.67	51,344.09–55,552.87	<0.001
Number of coded opportunistic or AIDS-defining conditions	Q25	1,759.45	235.97	1,296.94–2,221.96	<0.001
	Q50	2,282.38	284.81	1,724.15–2,840.62	<0.001
	Q75	3,130.07	330.26	2,482.77–3,777.37	<0.001
	Q90	3,674.98	413.48	2,864.57–4,485.39	<0.001

Note: N=2,469 hospitalization episodes. Heteroskedasticity-robust standard errors are reported. All covariates in the primary conditional quantile regression models are displayed. Female sex, low resource-use intensity, employee medical insurance, local care seeking, and no prolonged length of stay were reference groups.
